# Comorbidities associated with COVID-19 mortality in adults in Lima, Peru: a retrospective cohort study

**DOI:** 10.17843/rpmesp.2023.402.12170

**Published:** 2023-06-30

**Authors:** M. Gabriela Soto-Cabezas, Mary F. Reyes-Vega, Anderson N. Soriano-Moreno, Luis Ordoñez-Ibargüen, Kevin S. Martel, Noemi Flores-Jaime, Jenny Chirinos-Saire, J. Pierre Velásquez, Cesar V. Munayco

**Affiliations:** 1 Centro Nacional de Epidemiología, Prevención y Control de Enfermedades, Ministerio de Salud. Lima, Peru Centro Nacional de Epidemiología, Prevención y Control de Enfermedades Ministerio de Salud Lima Peru

**Keywords:** COVID-19, Comorbidity, Prognosis, Hospitalization, Peru

## Abstract

**Objectives.:**

To evaluate comorbidities associated with mortality in adult patients hospitalized due to COVID-19 in hospitals in Lima and Callao.

**Materials and methods.:**

In this retrospective cohort study, we analyzed data from adult patients hospitalized due to COVID-19 reported to the National Epidemiological Surveillance System of the Peruvian Ministry of Health from March to October 2020. We estimated relative risks with 95% confidence intervals using Poisson regression models with robust variance to assess comorbidities associated with mortality by age group: young adults (18-29 years), adults (30-59 years) and older adults (≥60 years).

**Results.:**

We included 2366 young adults, 23,781 adults and 25,356 older adults. Older adults had the highest mortality (63.7%) compared to adults (27.1%) and young adults (8.5%). Regardless of age group, the presence of neurological disease, renal disease, liver disease, and cancer was associated with an increased risk of mortality. Additionally, cardiovascular disease was also a risk factor in young adults; obesity, diabetes, cardiovascular disease, chronic lung disease, and immunodeficiency in adults; and obesity and chronic lung disease in the elderly.

**Conclusions.:**

Regardless of age groups, individuals with chronic neurologic disease, renal disease, liver disease, and cancer were at high risk of death from COVID-19.

## INTRODUCTION

In Peru, the first wave of the 2019 coronavirus disease pandemic (COVID-19) began on March 6, 2020. Since then, Peru has experienced five waves with more than four million people infected, of which almost three hundred thousand required hospitalization ^1^. COVID-19 took the Peruvian health system by surprise and caused the collapse of hospitals right at the beginning of the pandemic. Nationally, there were only about one hundred intensive care beds and oxygen volume production was limited ^2^. The government responded to this situation with several measures, including restricting mobilization and reinforcing the health system ^3^. Within four months, the number of intensive care beds was increased to almost 1000 ^2^. However, the number of cases increased again in November, triggering a second wave, with almost two million cumulative cases and almost 200,000 deaths ^1^.

The clinical spectrum of SARS-CoV-2 infection ranges from asymptomatic infection to severe disease; the highest percentage of cases are asymptomatic, mild or moderate, but there is a group of patients who develop severe disease, require hospital management and have a higher risk of death ^4^. Studies in different populations, have shown that the following are risk factors for mortality from COVID-19: older age, male sex, comorbidities such as obesity, cardiovascular disease, cancer, chronic kidney disease, immunosuppression, as well as clinical factors such as oxygen saturation at hospital admission, elevated creatinine and lactate dehydrogenase (LDH) levels ^5^^-^^8^. In addition, the collapse of the healthcare system also played a crucial role in mortality.

Understanding the main factors that increase the risk of death is crucial to improve the health system response. Few studies have investigated the factors associated with mortality in different age groups. In this study, we sought to evaluate comorbidities associated with mortality in adult patients hospitalized in the provinces of Lima and Callao during the first wave of COVID-19 in Peru.

**KEY MESSAGES**

Motivation for the study. During the COVID-19 pandemic, the mortality rate from this disease was higher in adults and the elderly. Therefore, it is important to identify the factors that were associated with mortality from COVID-19 in adults, by age group.

Main findings. Chronic neurological disease, kidney disease, liver disease, and cancer increased the risk of dying from COVID-19 in the three age groups we analyzed, which were made up of hospitalized patients from Lima and Callao. The risk of mortality associated with comorbidities was higher in patients aged 18 to 29.

Implications. This study helps to identify the groups of patients with the highest risk of death from COVID-19, according to age group and type of comorbidity.

## MATERIALS AND METHODS

### Study design and data sources

We conducted a retrospective cohort study, using data from the National Epidemiological Surveillance System of the Peruvian Ministry of Health (NotiWeb) ^11^, which collects clinical and epidemiological data on different notifiable infectious diseases, and from the National Mortality Agency (SINADEF) database, which collects data from death certificates ^12^. The national identity document (DNI) or immigration card (CE) was used as a unique identifier to unite the two databases, once consolidated, the data was encrypted using a process developed by the General Office of Information Technologies (OGTI) of the Ministry of health. Subsequently, the nominal data was eliminated, leaving only a generated identifier. The person in charge of carrying out this process was JPVR (author). For the analysis, only the consolidated database with the encrypted identifiers was shared.

### Study population

We analyzed all cases reported during the first wave of the COVID-19 pandemic in Peru (from March 6 to October 31, 2020). We included patients older than 18 years with a confirmed diagnosis of COVID-19 (positive molecular PCR or reactive IgM/IgG rapid test) ^9^, who were hospitalized in a health facility. Pregnant and postpartum women were excluded from the analysis.

### Dependent and independent variable

Mortality was the dependent variable. Deceased patients were considered as those who were registered in the SINADEF database from March 6 to September 10, 2021 and whose cause of death was one of the following ICD-10 codes: B972 (coronavirus as cause of diseases classified in other chapters), U071 (acute respiratory illness due to the new coronavirus) or U072 (COVID-19, unidentified virus). If the case did not meet this criterion, it was defined as a survivor. The independent variables were the comorbidities registered in the epidemiological record. These included obesity, diabetes, cardiovascular disease, chronic neurological disease, kidney disease, chronic lung disease, asthma, liver disease, immunodeficiency, and cancer.

### Covariates

We also included other variables from the epidemiological records. The demographic variables included age in years, sex (male, female), and health worker status (no, yes). We defined the age groups according to the classification of the Ministry of Health: youth (18-29 years), adults (30-59 years) and older adults (≥60 years) ^10^. Other variables were admission to the intensive care unit (ICU), the need for mechanical ventilation support and the type of health facility (EESS) with four categories: Ministry of Health (MINSA), Social Health Security (EsSalud), Armed Forces and National Police (FFAA/PNP) and private institutions. The time in which the case was reported was divided into four periods (March/April, May/June, July/August, September/October).

### Statistical analysis

Categorical variables are displayed using absolute and relative frequencies.

We used chi-square tests to compare characteristics between age groups, as well as to compare the characteristics between deceased and survivors in each of the age groups. To assess comorbidities associated with mortality, we estimated crude and adjusted relative risks (RR) with their confidence intervals (95% CI) using Poisson regression with robust variance. The regression analyzes were performed while stratifying by age group. The adjusted model included the following variables: age in years, sex, being a health worker, admission to intensive care, need for mechanical ventilation, type of health facility, and the period in which the case was reported, based on epidemiological criteria. A value of p<0.05 was considered statistically significant. Data was cleaned and analyzed in R, version 4.0.3 (R Foundation for Statistical Computing).

### Ethical aspects

This study used secondary databases of the National Epidemiological Surveillance System of the Ministry of Health of Peru; the databases we used were completely anonymized. This study was approved by the CDC - Peru for its publication (file No. 21-145573-001). The study was also registered on the Health Research Projects Registry (PRISA) portal, with the code: EI00000002704.

## RESULTS

### Population characteristics

**Figure 1:**
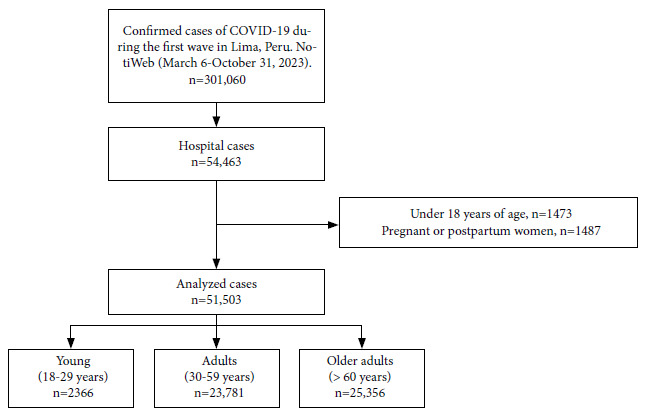
Flow chart of study participants.

We included 51,503 confirmed cases of COVID-19 that were reported to the epidemiological surveillance system as hospitalized in the provinces of Lima and Callao during the first wave of the COVID-19 pandemic in Peru, who met the inclusion criteria described in Figure 1. Most of the cases were adults (46.2%) or older adults (49.2%); 60.4% were male and most were hospitalized in MINSA (39.5%) or EsSalud (40.4%) health establishments. Comorbidities were reported in 29.2% of the patients. The most frequent comorbidities were cardiovascular disease (15.6%), diabetes (11.1%), and obesity (5.14%); 7.9% were admitted to intensive care and 6.6% used mechanical ventilation. When comparing by age groups, we found that comorbidities were present in 8.8%, 21.9%, and 37.9% of the young, adult, and older adult cases, respectively (p<0.001). The prevalence of comorbidities increased in relation to the age groups (p<0.050), with the exception of obesity, asthma and immunodeficiencies, which were more frequent among the adult population. Older adults died more frequently (63.7%) compared to adults (27.1%) and young people (8.5%, p<0.001). The frequency of ICU admission and use of mechanical ventilation also increased with age (Table 1).

**Table 1:** General and clinical characteristics of patients with confirmed COVID-19 who were hospitalized in health facilities in Lima, Peru. March to October 2020.

Factors	Total N=51,503	Age groups			p-value ^a^
		Young (18-29)	Adults (30-59)	Older adults (≥60)	
		n=2366 (4.6%)	n=23,781 (46.2%)	n=25,356 (49.2%)	
	n (%)	n (%)	n (%)	n (%)	
Sex					<0.001
Male	31,104 (60.4)	939 (39.7)	15,099 (63.5)	15,066 (59.4)	
Female	20,399 (39.6)	1427 (60.3)	8682 (36.5)	10,290 (40.6)	
Healthcare worker					<0.001
No	46,601 (90.5)	2132 (90.1)	21,144 (88.9)	23,325 (92.0)	
Yes	4902 (9.5)	234 (9.9)	2637 (11.1)	2031 (8.0)	
Comorbidities	15,026 (29.2)	208 (8.8)	5219 (21.9)	9599 (37.9)	<0.001
Obesity	2649 (5.1)	95 (4.0)	1570 (6.6)	984 (3.9)	<0.001
Diabetes	5737 (11.1)	30 (1.3)	1944 (8.2)	3763 (14.8)	<0.001
Cardiovascular disease	8059 (15.6)	26 (1.1)	1814 (7.6)	6219 (24.5)	<0.001
Chronic neurological disease	536 (1.0)	12 (0.5)	138 (0.6)	386 (1.5)	<0.001
Kidney disease	1144 (2.2)	19 (0.8)	324 (1.4)	801 (3.2)	<0.001
Chronic pulmonary disease	847 (1.6)	16 (0.7)	244 (1.03)	587 (2.3)	<0.001
Asthma	637 (1.2)	31 (1.3)	351 (1.5)	255 (1.0)	0.001
Liver disease	279 (0.5)	6 (0.3)	89 (0.4)	184 (0.7)	<0.001
Immunodeficiency	157 (0.3)	8 (0.3)	98 (0.4)	51 (0.2)	0.004
Cancer	757 (1.5)	15 (0.6)	243 (1.0)	499 (2.0)	<0.001
Type of health establishment					<0.001
MINSA	20,353 (39.5)	1471 (62.2)	9584 (40.3)	9298 (36.7)	
EsSalud	20,830 (40.4)	454 (19.2)	8260 (34.7)	12,116 (47.8)	
FFAA/PNP	3368 (6.6)	122 (5.1)	1649 (7.0)	1597 (6.3)	
Private	6952 (13.5)	319 (13.5)	4288 (18.0)	2345 (9.2)	
Admission to ICU	3403 (6.6)	58 (2.5)	1748 (7.4)	1597 (6.3)	<0.001
Use of mechanical ventilation	4073 (7.9)	83 (3.5)	2152 (9.1)	1838 (7.2)	<0.001
Infection period					<0.001
March/April, 2020	5911 (11.5)	204 (8.6)	3072 (12.9)	2635 (10.4)	
June/July, 2020	19,580 (38.0)	899 (38.0)	9033 (38.0)	9648 (38.0)	
August/September, 2020	19,027 (36.9)	816 (34.5)	8688 (36.5)	9523 (37.6)	
October/November, 2020	6985 (13.6)	447 (18.9)	2988 (12.6)	3550 (14.0)	
Deceased	22,787 (44.2)	200 (8.5)	6445 (27.1)	16,142 (63.7)	<0.001

a p-value calculated with the chi-square test.

MINSA: Ministry of Health; EsSalud: Peruvian Social Health Insurance; FFAA/PNP: Armed Forces and National Police; ICU: intensive care unit.

### Characteristics according to age group and mortality

Males died more frequently than females (p<0.001). Mortality was significantly lower in cases hospitalized in a private establishment compared to establishments linked to public services (p<0.001). Mortality was lower in adults who were health workers (23.1%) compared to those who were not (27.6%, p<0.001). Mortality was higher in cases with comorbidities, especially among young people. Young people with some comorbidity had three times (7.2% vs. 21.6%) the probability of dying than those young patients without comorbidities (p<0.001). Adult and older adult patients with comorbidities also died more frequently than those without comorbidities, but the difference was smaller than in younger patients. Both in the group of young people, adults and the elderly, cases with cardiovascular disease, chronic neurological disease, kidney disease, liver disease or cancer had a higher frequency of mortality compared to those without these conditions (p<0.05) (Table 2).

**Table 2:** Comparison of characteristics between deceased and survivor patients with confirmed 2019 coronavirus disease admitted to hospitalization in health facilities in Lima, Peru. March to October 2020.

Factors	Young (18-29)		p-value ^a^	Adults (30-59)		p-value ^a^	Older adults (≥60)		p-value ^a^
	Survivors n=2166	Deceased n=200		Survivors n=17,336	Deceased n=6445		Survivors n=9214	Deceased n=16,142	
	n (%)	n (%)		n (%)	n (%)		n (%)	n (%)	
Sex			<0.001			<0.001			<0.001
Female	1347 (94.4)	80 (5.6)		6682 (77.0)	2000 (23.0)		4144 (40.3)	6146 (59.7)	
Male	819 (87.2)	120 (12.8)		10 654 (70.6)	4445 (29.4)		5070 (33.7)	9996 (66.3)	
Health worker			0.417			<0.001			0.756
No	1948 (91.4)	184 (8.6)		15 308 (72.4)	5836 (27.6)		8469 (36.3)	14 856 (63.7)	
Yes	218 (93.2)	16 (6.8)		2028 (76.9)	609 (23.1)		745 (36.7)	1286 (63.3)	
Comorbidities			<0.001			<0.001			<0.001
No	2003 (92.8)	155 (7.2)		14 022 (75.5)	4540 (24.5)		5935 (37.7)	9822 (62.3)	
Yes	163 (78.4)	45 (21.6)		3314 (63.5)	1905 (36.5)		3279 (34.2)	6320 (65.8)	
Obesity			0.192			<0.001			0.018
No	2083 (91.7)	188 (8.3)		16,387 (73.8)	5824 (26.2)		8892 (36.5)	15,480 (63.5)	
Yes	83 (87.4)	12 (12.6)		949 (60.4)	621 (39.6)		322 (32.7)	662 (67.3)	
Diabetes			0.036			<0.001			0.684
No	2142 (91.7)	194 (8.3)		16,072 (73.6)	5765 (26.4)		7835 (36.3)	13,758 (63.7)	
Yes	24 (80.0)	6 (20.0)		1264 (65.0)	680 (35.0)		1379 (36.6)	2384 (63.4)	
Cardiovascular disease			<0.001			<0.001			<0.001
No	2150 (91.9)	190 (8.1)		16,173 (73.6)	5794 (26.4)		7089 (37.0)	12,048 (63.0)	
Yes	16 (61.5)	10 (38.5)		1163 (64.1)	651 (35.9)		2125 (34.2)	4094 (65.8)	
Chronic neurological disease			<0.001			<0.001			<0.001
No	2160 (91.8)	194 (8.2)		17,255 (73.0)	6388 (27.0)		9120 (36.5)	15,850 (63.5)	
Yes	6 (50.0)	6 (50.0)		81 (58.7)	57 (41.3)		94 (24.4)	292 (75.6)	
Kidney disease			<0.001			<0.001			<0.001
No	2157 (91.9)	190 (8.1)		17,188 (73.3)	6269 (26.7)		9006 (36.7)	15,549 (63.3)	
Yes	9 (47.4)	10 (52.6)		148 (45.7)	176 (54.3)		208 (26.0)	593 (74.0)	
Chronic lung disease			0.640			<0.001			<0.001
No	2152 (91.6)	198 (8.4)		17,188 (73.0)	6349 (27.0)		9066 (36.6)	15,703 (63.4)	
Yes	14 (87.5)	2 (12.5)		148 (60.7)	96 (39.3)		148 (25.2)	439 (74.8)	
Asthma			0.742			0.199			0.305
No	2138 (91.6)	197 (8.4)		17,069 (72.9)	6361 (27.1)		9113 (36.3)	15,988 (63.7)	
Yes	28 (90.3)	3 (9.7)		267 (76.1)	84 (23.9)		101 (39.6)	154 (60.4)	
Liver disease			0.085			<0.001			0.005
No	2162 (91.6)	198 (8.4)		17,287 (73.0)	6405 (27.0)		9166 (36.4)	16,006 (63.6)	
Yes	4 (66.7)	2 (33.3)		49 (55.1)	40 (44.9)		48 (26.1)	136 (73.9)	
Immunodeficiency			0.507			<0.001			0.554
No	2159 (91.6)	199 (8.4)		17,291 (73.0)	6392 (27.0)		9198 (36.3)	16,107 (63.7)	
Yes	7 (87.5)	1 (12.5)		45 (45.9)	53 (54.1)		16 (31.4)	35 (68.6)	
Cancer			<0.001			<0.001			<0.001
No	2162 (92.0)	189 (8.0)		17,234 (73.2)	6304 (26.8)		9098 (36.6)	15,759 (63.4)	
Yes	4 (26.7)	11 (73.3)		102 (42.0)	141 (58.0)		116 (23.2)	383 (76.8)	
Type of health facility			<0.001			<0.001			<0.001
MINSA	1353 (92.0)	118 (8.02)		6237 (65.1)	3347 (34.9)		3088 (33.2)	6210 (66.8)	
EsSalud	392 (86.3)	62 (13.7)		5891 (71.3)	2369 (28.7)		4113 (33.9)	8003 (66.1)	
FFAA/PNP	109 (89.3)	13 (10.7)		1259 (76.3)	390 (23.7)		533 (33.4)	1064 (66.6)	
Private	312 (97.8)	7 (2.2)		3949 (92.1)	339 (7.9)		1480 (63.1)	865 (36.9)	
Admission to ICU			<0.001			<0.001			<0.001
No	2134 (92.5)	174 (7.5)		16,679 (75.7)	5354 (24.3)		8930 (37.6)	14,829 (62.4)	
Yes	32 (55.2)	26 (44.8)		657 (37.6)	1091 (62.4)		284 (17.8)	1313 (82.2)	
Use of mechanical ventilation			<0.001			0.000			<0.001
No	2122 (92.9)	161 (7.1)		16,563 (76.6)	5066 (23.4)		8900 (37.8)	14,618 (62.2)	
Yes	44 (53.0)	39 (47.0)		773 (35.9)	1379 (64.1)		314 (17.1)	1524 (82.9)	
Diagnostic period			0.182			<0.001			<0.001
March/April, 2020	179 (87.7)	25 (12.3)		2242 (73.0)	830 (27.0)		990 (37.6)	1645 (62.4)	
June/July, 2020	821 (91.3)	78 (8.7)		6307 (69.8)	2726 (30.2)		3150 (32.6)	6498 (67.4)	
August/September,2020	754 (92.4)	62 (7.6)		6361 (73.2)	2327 (26.8)		3558 (37.4)	5965 (62.6)	
October/November, 2020	412 (92.2)	35 (7.8)		2426 (81.2)	562 (18.8)		1516 (42.7)	2034 (57.3)	

a p-value calculated with the chi-square test.

MINSA: Ministry of Health; EsSalud: Peruvian Social Health Insurance; FFAA/PNP: Armed Forces and National Police; ICU: intensive care unit.

### Comorbidities associated with mortality

In the adjusted regression analysis, we found that neurological disease, kidney disease, liver disease, and cancer were associated with a higher risk of mortality regardless of age group. Additionally, cardiovascular disease (RR=2.16; 95% CI: 1.24-3.74) was associated with a higher risk of mortality in young patients. In adults, obesity (RR=1.30; 95% CI: 1.22-1.39), diabetes (RR=1.12; 95% CI: 1.06-1.20), cardiovascular disease (RR=1.14; 95% CI: 1.07-1.21), chronic lung disease (RR=1.30; 95% CI: 1.1-1.51) and immunodeficiency (RR=2 .18; 95% CI: 1.77-2.69) were also comorbidities associated with mortality. Obesity (RR=1.12; 95% CI: 1.07-1.17) and chronic lung disease (RR=1.13; 95% CI: 1.07-1.18) were also associated with mortality in older patients. Asthma was not associated with an increased risk of mortality in the bivariate or multivariate analysis for any of the age groups (Table 3).

**Table 3:** Poisson regression analysis with robust variance for comorbidities associated with in-hospital mortality in patients with COVID-19 admitted to hospitalization in Lima, Peru. March to October 2020.

Comorbidities	Crude model			Adjusted model ^a^		
	RR	95% CI	p-value	RR	95% CI	p-value
Age 18-29 years (n=2366)						
Obesity	1.53	0.88-2.63	0.130	0.75	0.45-1.26	0.282
Diabetes	2.41	1.16-4.99	0.018	1.46	0.72-2.96	0.296
Cardiovascular disease	4.74	2.86-7.85	<0.001	2.16	1.24-3.74	0.006
Chronic neurological disease	6.07	3.39-10.85	<0.001	5.03	2.60-9.74	<0.001
Renal disease	6.50	4.15-10.17	<0.001	5.01	2.88-8.71	<0.001
Chronic lung disease	1.48	0.40-5.46	0.553	0.86	0.31-2.40	0.777
Asthma	1.15	0.39-3.39	0.804	0.51	0.24-1.10	0.087
Liver disease	3.97	1.27-12.42	0.018	3.82	1.16-12.55	0.027
Immunodeficiency	1.48	0.24-9.31	0.675	1.02	0.16-6.61	0.986
Cancer	9.12	6.53-12.74	<0.001	7.29	4.56-11.64	<0.001
Age 30-59 years (n=23,781)						
Obesity	1.51	1.41-1.61	<0.001	1.30	1.22-1.39	<0.001
Diabetes	1.32	1.24-1.41	<0.001	1.12	1.06-1.20	<0.001
Cardiovascular disease	1.36	1.27-1.45	<0.001	1.14	1.07-1.21	<0.001
Chronic neurological disease	1.53	1.25-1.87	<0.001	1.36	1.12-1.65	0.002
Kidney disease	2.03	1.84-2.25	<0.001	1.87	1.68-2.09	<0.001
Chronic lung disease	1.46	1.25-1.71	<0.001	1.30	1.11-1.51	0.001
Asthma	0.88	0.73-1.06	0.188	0.92	0.77-1.10	0.378
Liver disease	1.66	1.32-2.09	<0.001	1.55	1.21-1.99	0.001
Immunodeficiency	2.00	1.67-2.41	<0.001	2.18	1.77-2.69	<0.001
Cancer	2.17	1.94-2.42	<0.001	2.38	2.10-2.70	<0.001
Age >60 years (n=25,356)						
Obesity	1.06	1.01-1.11	0.011	1.12	1.07-1.17	<0.001
Diabetes	0.99	0.97-1.02	0.672	1.00	0.98-1.03	0.859
Cardiovascular disease	1.05	1.02-1.07	<0.001	0.99	0.96-1.01	0.179
Chronic neurological disease	1.19	1.13-1.26	<0.001	1.10	1.03-1.16	0.002
Kidney disease	1.17	1.12-1.22	<0.001	1.10	1.06-1.15	<0.001
Chronic lung disease	1.18	1.12-1.24	<0.001	1.13	1.07-1.18	<0.001
Asthma	0.95	0.86-1.05	0.296	0.98	0.89-1.07	0.613
Liver disease	1.16	1.07-1.27	0.001	1.20	1.10-1.31	<0.001
Immunodeficiency	1.08	0.90-1.30	0.427	1.14	0.96-1.37	0.144
Cancer	1.21	1.15-1.27	<0.001	1.23	1.17-1.29	<0.001

a Adjusted for age, sex in years, being health personnel, admission to intensive care, need for mechanical ventilation, type of health facility and the period in which the case was reported. RR: relative risk, 95% CI: 95% confidence interval.

## DISCUSSION

In this study, we analyzed data from adult patients with COVID-19 who were hospitalized during the first wave of the pandemic in Lima, Peru. We found that the frequency of comorbidities and mortality increased with age. Chronic neurological disease, kidney disease, liver disease, and cancer were associated with mortality, regardless of the age group. The risk of mortality associated with comorbidities was higher in young adults compared to adults and older adults. To our knowledge, this is the first study to assess comorbidities associated with mortality in different age groups. Previous studies in Peru have reported that oxygen saturation on admission ^6^^,^^13^, being over 65 years of age, lactate dehydrogenase greater than 720 U/L ^13^ and the use of mechanical ventilation or admission to the ICU ^14^ were factors associated with mortality.

The mortality rate in Lima and Callao was higher compared to the world average, estimated at 15% by a systematic review ^15^, but similar to other Latin American countries such as Colombia (40%) ^(^^16^. Different factors can explain why Peru has one of the highest mortality rates in the world. A contributing factor could be the collapse of the health system due to the rapid occupation of hospitalization and critical care beds, the lack of oxygen and mechanical ventilation equipment ^17^ and the high rates of self-medication with antibiotics or anti-inflammatories among the general population, which was estimated to be around 39% among the residents of Lima ^18^.

Patients older than 59 years had a mortality rate 20-times higher than those aged 18 to 29 years. Young adults had a mortality rate similar to what was reported by a study in the United States ^19^. Older adults had higher mortality rates compared to other high-income countries (35.5%) ^20^, but similar to studies conducted in Latin American countries such as Brazil (64%) ^21^ and Mexico (69%). ^22^. Age is one of the independent risk factors for mortality, identified since the beginning of the pandemic ^23^. Older people have a lower functional reserve and higher levels of angiotensin-converting enzyme 2 (ACE2), which may play a key role in the multiorgan involvement caused by SARS-CoV-2 ^24^. Interestingly, we found that the risk of mortality associated with comorbidities was higher in young patients (18-29 years) compared to older patients. Younger patients with comorbidities died three times more than young patients without comorbidities, a difference much greater than what was found in older patients. Regression models showed greater association between comorbidities and mortality in younger patients than in older patients. One potential explanation is that young patients may have more severe or complex comorbidities than older patients leading to a worse prognosis when contracting COVID-19 and suggests that young people with comorbidities may require particularly close monitoring as high-risk group.

Chronic neurological disease, kidney disease, liver disease, and cancer were associated with mortality, regardless of age group. This finding is consistent with previous research. For example, a study in Spain found that patients with pre-existing neurological comorbidities were 1.76 times more likely to die, regardless of bed occupancy or the type of treatment ^25^. This could be explained by the baseline health status of these patients; patients with chronic neurological disease have greater frailty and less reserve than people without chronic neurological disease. Neurological manifestations during acute illness have also been associated with a worse prognosis ^26^. A systematic review showed that patients with chronic kidney disease are seven times more likely to die than patients without pre-existing disease ^27^. Patients with kidney disease are in a proinflammatory state and have functional defects in innate and acquired immune cells, which increases vulnerability to infection and also worsens the prognosis ^28^. Several studies have also shown that patients with chronic kidney disease are at increased risk of pneumonia and severe pneumonia ^29^. Liver disease has also been associated with severity and mortality ^30^. Obesity was also a risk factor for the group of patients over 30 years of age, which is consistent with a meta-analysis that reported that obese patients had up to four times more mortality ^31^. We found no association regarding cases between 18 and 29 years of age. We did not find an association between asthma and an increased risk of mortality. This result also agrees with previous articles. The WHO conducted a systematic review that concluded that the role of asthma as a risk factor for death from COVID-19 remains unclear ^32^. Another review found that the risk of infection was lower in people with asthma compared to non-asthmatics, but found no difference in terms of hospitalization, intensive care admission, ventilator use, or mortality ^33^.

Our study has some limitations. First, this is a retrospective analysis of secondary data (mainly from the epidemiological surveillance system) that was not designed to estimate risk factors. For this reason, we could not include as many clinical details as an electronic medical record would. We found a lower prevalence of obesity ^34^, chronic obstructive pulmonary disease ^35^ and kidney disease ^36^ than studies with specialized tests, which could be due to the lack of standardized epidemiological definitions of comorbidities. We were also unable to explore the severity of comorbidities, which may modify the effect on mortality. Our findings should be cautiously applied to other populations due to the unique chaotic situation experienced during the first wave of the pandemic in Peru, which may not reflect the reality of all hospitalized COVID-19 patients. We do not have data on variables such as oxygen saturation on admission or the treatment received during hospitalization, which could play an important role in the mortality rates. Some patients were diagnosed only with rapid serological tests. These tests have a lower diagnostic performance than molecular tests, so it is possible that some patients had false positives and false negatives. On the other hand, this study has the strength of analyzing a broad and representative sample of hospitalized patients. Previous studies in local settings have not explored comorbidities associated with mortality, so our results complement the study of COVID-19 in Peru. Furthermore, this is the first study to evaluate associated comorbidities by age group. Our multivariate analysis considered possible confounding factors, such as hospital ICU bed occupancy, type of health facility, and time from symptom onset to hospitalization.

In conclusion, in this retrospective cohort of hospitalized patients with COVID-19, we found evidence that chronic neurological disease, kidney disease, liver disease, and cancer were comorbidities associated with mortality regardless of age group. Furthermore, despite the fact that patients older than 60 years had higher mortality compared with younger patients, the risk of mortality associated with comorbidities was higher in young adults.
