# Supplementary material for: Comorbidities associated with COVID-19 mortality in adults in Lima, Peru: a retrospective cohort study
Source: Rev Peru Med Exp Salud Publica. 2023 Jun 30;40(2):132–40. doi: 10.17843/rpmesp.2023.402.12170 (PMC10953670; doi:10.17843/rpmesp.2023.402.12170)
Supplement: Translation

# Translation: Comorbilidades asociadas a la mortalidad por COVID-19 en adultos en Lima, Perú: un estudio de cohorte retrospectiva

**Authors:** M. Gabriela Soto-Cabezas, Mary F. Reyes-Vega, Anderson N. Soriano-Moreno, Luis Ordoñez-Ibargüen, Kevin S. Martel, Noemi Flores-Jaime, Jenny Chirinos-Saire, J. Pierre Velásquez, Cesar V. Munayco

**Affiliations:** 1 Centro Nacional de Epidemiología, Prevención y Control de Enfermedades, Ministerio de Salud. Lima, Perú.

**Keywords:** COVID-19, Comorbilidades, Pronóstico, Hospitalización, Perú

## Abstract

**Objetivos.:**

Evaluar las comorbilidades asociadas a la mortalidad en pacientes adultos hospitalizados por COVID-19 de diferentes grupos de edad en hospitales de Lima y Callao.

**Materiales y métodos.:**

En este estudio de cohorte retrospectiva analizamos datos de pacientes adultos hospitalizados por COVID-19, notificados al Sistema Nacional de Vigilancia Epidemiológica del Ministerio de Salud de Perú de marzo a octubre del 2020. Se estimaron riesgos relativos con intervalos de confianza al 95% mediante modelos de regresión de Poisson con varianza robusta para evaluar las comorbilidades asociadas a la mortalidad por grupos de edad: jóvenes (18-29 años), adultos (30-59 años) y mayores (≥60 años).

**Resultados.:**

Se incluyeron 2366 jóvenes, 23781 adultos y 25356 adultos mayores en el análisis. Los adultos mayores presentaron la mortalidad más alta (63,7%) en comparación con adultos (27,1%) y jóvenes (8,5%). Independientemente del grupo de edad, la presencia de enfermedad neurológica, enfermedad renal, enfermedad hepática y cáncer se asoció a un mayor riesgo de mortalidad. Adicionalmente, la enfermedad cardiovascular fue también un factor de riesgo en los jóvenes; la obesidad, la diabetes, la enfermedad cardiovascular, la enfermedad pulmonar crónica y la inmunodeficiencia en los adultos; y la obesidad y la enfermedad pulmonar crónica en los mayores.

**Conclusiones:**

Independientemente de los grupos de edad, los individuos con enfermedad neurológica crónica, enfermedad renal, enfermedad hepática y cáncer tendrían un alto riesgo de morir por la COVID-19.

## INTRODUCCIÓN

En el Perú, la primera ola de la pandemia de la enfermedad por coronavirus 2019 (COVID-19) comenzó el 6 de marzo del 2020. Desde entonces, Perú ha experimentado cinco oleadas con más de cuatro millones de personas infectadas, de las cuales casi trescientos mil requirieron hospitalización ^1^. La COVID-19 tomó por sorpresa al sistema de salud peruano y provocó el colapso de los hospitales justo al comienzo de la pandemia. A nivel nacional, solo había alrededor de cien camas de cuidados intensivos y la producción de volumen de oxígeno era limitada ^2^. El gobierno respondió a esta situación con varias medidas, entre ellas la restricción de la movilización y el refuerzo del sistema de salud ^3^. En cuestión de cuatro meses, se incrementó el número de camas de cuidados intensivos a casi mil ^2^. Sin embargo, el número de casos volvió a aumentar en noviembre, desencadenando una segunda ola, con casi dos millones de casos acumulados y casi 200 000 muertes ^1^.

El espectro clínico de la infección por SARS-CoV-2 va desde una infección asintomática hasta una enfermedad grave, el mayor porcentaje de casos se presentan de forma asintomática, leve o moderada, pero existen un grupo de pacientes que desarrollan enfermedad severa, que requiere manejo hospitalario y tiene mayor riesgo de muerte ^4^. Estudios realizados en diferentes poblaciones, han demostrado que los factores de riesgo de la mortalidad por la COVID-19 son una mayor edad, el sexo masculino, las comorbilidades como la obesidad, las enfermedades cardiovasculares, el cáncer, la enfermedad renal crónica, la inmunosupresión, así como factores clínicos como la saturación de oxígeno al ingreso en el hospital, niveles elevados de creatinina y lactato deshidrogenasa (LDH) son consistentemente factores asociados con la mortalidad ^5^^-^^8^. Además, el colapso del sistema sanitario también jugó un papel crucial en la mortalidad.

Comprender los principales factores que incrementan el riesgo de muerte es crucial para mejorar la respuesta del sistema de salud. Pocos estudios han investigado los factores asociados a la mortalidad en los diferentes grupos de edad. En este estudio buscamos evaluar las comorbilidades asociadas a la mortalidad en pacientes adultos hospitalizados en las provincias de Lima y Callao durante la primera ola de la COVID-19 en el Perú.

**MENSAJE CLAVE**

Motivación para realizar el estudio. En la pandemia de la COVID-19, se observó que la mortalidad por esta enfermedad afectaba principalmente a los adultos y adultos mayores. Por lo que, es importante conocer qué factores se asocian a la mortalidad por la COVID-19 en los adultos, según grupo de edad.

Principales hallazgos. En los tres grupos de edad analizados, la enfermedad neurológica crónica, la enfermedad renal, la enfermedad hepática y el cáncer conllevan a un alto riesgo de morir por la COVID-19, en pacientes hospitalizados en Lima y Callao. El riesgo de mortalidad asociada con comorbilidades fue mayor en pacientes de 18 a 29 años.

Implicancias. Este estudio contribuye a identificar los grupos de pacientes con el mayor riesgo de muerte por la COVID-19, según grupo de edad y tipo de comorbilidad.

## MATERIALES Y MÉTODOS

### Diseño de estudio y fuentes de datos

Realizamos un estudio de cohorte retrospectivo, utilizando datos del Sistema Nacional de Vigilancia Epidemiológica del Ministerio de Salud de Perú (NotiWeb) ^11^, que recopila datos clínicos y epidemiológicos de diferentes enfermedades infecciosas de notificación obligatoria, y de la base de datos del sistema nacional de mortalidad (SINADEF), que recopila datos de los certificados de defunción ^12^. Se utilizó el documento nacional de identidad (DNI) o carnet de extranjería (CE) como identificador único para unir las dos bases, una vez consolidada se aplicó un proceso de encriptación desarrollado por la Oficina General de Tecnologías de la Información (OGTI) del Ministerio de Salud. Posteriormente los datos nominales fueron eliminados, quedando únicamente un identificador generado. La persona encargada de realizar este proceso fue JPVR (autor). Para el análisis, únicamente se compartió la base consolidada con los identificadores encriptados.

### Población de estudio

Se analizaron a todos los casos notificados durante la primera ola de la pandemia de la COVID-19 en Perú (del 6 de marzo al 31 de octubre del 2020). Incluimos a pacientes mayores de 18 años con diagnóstico confirmado de COVID-19 (PCR molecular positivo o prueba rápida de IgM/IgG reactiva) ^9^, que se hospitalizaron en un establecimiento de salud. Se excluyeron gestantes y puérperas del análisis.

### Variable dependiente e independiente

La variable dependiente fue la mortalidad. Definimos como fallecidos a aquellos pacientes que estuvieron registrados en la base del SINADEF desde el 6 de marzo hasta el 10 de setiembre del 2021 y que tuvieron como causa de fallecimiento uno los siguientes códigos CIE-10: B972 (coronavirus como causa de enfermedades clasificadas en otros capítulos), U071 (enfermedad respiratoria aguda debida al nuevo coronavirus) o U072 (COVID-19, virus no identificado). Si el caso no cumplió con este criterio fue definido como sobreviviente. Las variables independientes fueron las comorbilidades registradas en la ficha epidemiológica. Estas incluyeron a la obesidad, diabetes, enfermedad cardiovascular, enfermedad neurológica crónica, enfermedad renal, enfermedad pulmonar crónica, asma, enfermedad hepática, inmunodeficiencia y cáncer.

### Covariables

Analizamos también otras variables presentes en la ficha epidemiológica. Las variables demográficas incluyeron la edad en años, el sexo (masculino, femenino) y la condición de ser trabajador de la salud (no, sí). Definimos los grupos de edad según la clasificación del Ministerio de Salud: jóvenes (18-29 años), adultos (30-59 años) y adultos mayores (≥60 años) ^(^^10^. Otras variables fueron el ingreso a la unidad de cuidados intensivos (UCI) y la necesidad de soporte de ventilación mecánica y el tipo de establecimiento de salud (EESS) con cuatro categorías: Ministerio de Salud (MINSA), Seguro Social de Salud (EsSalud), Fuerzas Armadas y Policía Nacional (FFAA/PNP) e instituciones privadas. El período en el que se notificó el caso se dividió en cuatro intervalos (marzo/abril, mayo/junio, julio/agosto, septiembre/octubre).

### Análisis estadístico

Las variables categóricas fueron tabuladas utilizando frecuencias absolutas y relativas. Utilizamos las pruebas de chi cuadrado para comparar las características entre los grupos de edad; y también para comparar las características entre fallecidos y sobrevivientes en cada uno de los grupos de edad. Para evaluar las comorbilidades asociadas a la mortalidad estimamos riesgos relativos (RR) crudos y ajustados con sus intervalos de confianza (IC 95%) mediante regresión de Poisson con varianza robusta. Estos análisis de regresión se realizaron estratificando por grupo de edad. El modelo ajustado incluyó a la variable edad en años, el sexo, el ser trabajador de la salud, ingreso a cuidado intensivos, necesidad de ventilación mecánica, tipo de establecimiento de salud y el periodo en el que se notificó el caso, por criterio epidemiológico. Se consideró un valor de p < 0,05 como estadísticamente significativo. Realizamos la limpieza y el análisis de datos en R, versión 4.0.3 (R Foundation for Statistical Computing).

### Aspectos éticos

Este estudio utilizó bases de datos secundarias del Sistema Nacional de Vigilancia Epidemiológica del Ministerio de Salud de Perú, las bases utilizadas en el análisis fueron completamente anonimizadas. Este estudio contó con la aprobación del CDC - Perú para su publicación (expediente N° 21-145573-001). El estudio también fue registrado en el portal de Registro de Proyectos de Investigación en Salud (PRISA), con el código: EI00000002704.

## RESULTADOS

### Características de la población

Se analizaron 51503 casos de COVID-19 confirmados que fueron notificados al sistema de vigilancia epidemiológica como hospitalizados en las provincias de Lima y Callao durante la primera ola pandémica de la COVID-19 en Perú, que cumplieron los criterios de inclusión descritos en la Figura 1. La mayoría de los casos fueron adultos (46,2%) o adultos mayores (49,2%). El 60,4% fueron de sexo masculino y la mayoría fueron hospitalizados en establecimientos de salud del MINSA (39,5%) o EsSalud (40,4%). El 29,2% de los pacientes tuvieron alguna de las comorbilidades reportadas en la ficha epidemiológica. Las comorbilidades más frecuentes fueron la enfermedad cardiovascular (15,6%), la diabetes (11,1%) y la obesidad (5,14%). El 7,9% fue ingresado en cuidados intensivos y el 6,6% usó ventilación mecánica. Al comparar por grupos de edad observamos que las comorbilidades estuvieron presentes en el 8,8%, 21,9% y 37,9% de los casos jóvenes, adultos y adultos mayores, respectivamente (p<0,001). La prevalencia de comorbilidades se incrementó en relación con los grupos etarios (p<0,050), a excepción de la obesidad, el asma y las inmunodeficiencias, que fueron más frecuentes entre la población adulta. Los adultos mayores fallecieron con más frecuencia (63,7%) en comparación de los adultos (27,1%) y los jóvenes (8,5%, p<0,001). La frecuencia de admisión a UCI y de uso de ventilación mecánica también se incrementó con la edad (Tabla 1).

**Figura 1:** Diagrama de flujo de participantes en el estudio.

**Tabla 1:** Características generales y clínicas de los pacientes con enfermedad por coronavirus 2019 confirmada que ingresaron a hospitalización en establecimientos de salud de Lima, Perú. Marzo a octubre del 2020.

Factores	Total N=51 503	Grupos de edad			Valor de p ^a^
		Jóvenes (18-29)	Adultos (30-59)	Adultos mayores (≥60)	
		n=2366 (4,6%)	n=23 781 (46,2%)	n=25 356 (49,2%)	
	n (%)	n (%)	n (%)	n (%)	
Sexo					<0,001
Masculino	31 104 (60,4)	939 (39,7)	15 099 (63,5)	15 066 (59,4)	
Femenino	20 399 (39,6)	1427 (60,3)	8682 (36,5)	10 290 (40,6)	
Trabajador de salud					<0,001
No	46 601 (90,5)	2132 (90,1)	21 144 (88,9)	23 325 (92,0)	
Sí	4902 (9,5)	234 (9,9)	2637 (11,1)	2031 (8,0)	
Comorbilidades	15 026 (29,2)	208 (8,8)	5219 (21,9)	9599 (37,9)	<0,001
Obesidad	2649 (5,1)	95 (4,0)	1570 (6,6)	984 (3,9)	<0,001
Diabetes	5737 (11,1)	30 (1,3)	1944 (8,2)	3763 (14,8)	<0,001
Enfermedad cardiovascular	8059 (15,6)	26 (1,1)	1814 (7,6)	6219 (24,5)	<0,001
Enfermedad neurológica crónica	536 (1,0)	12 (0,5)	138 (0,6)	386 (1,5)	<0,001
Enfermedad renal	1144 (2,2)	19 (0,8)	324 (1,4)	801 (3,2)	<0,001
Enfermedad pulmonar crónica	847 (1,6)	16 (0,7)	244 (1,03)	587 (2,3)	<0,001
Asma	637 (1,2)	31 (1,3)	351 (1,5)	255 (1,0)	0,001
Enfermedad hepática	279 (0,5)	6 (0,3)	89 (0,4)	184 (0,7)	<0,001
Inmunodeficiencia	157 (0,3)	8 (0,3)	98 (0,4)	51 (0,2)	0,004
Cáncer	757 (1,5)	15 (0,6)	243 (1,0)	499 (2,0)	<0,001
Tipo de establecimiento de salud					<0,001
MINSA	20 353 (39,5)	1471 (62,2)	9584 (40,3)	9298 (36,7)	
EsSalud	20 830 (40,4)	454 (19,2)	8260 (34,7)	12 116 (47,8)	
FFAA/PNP	3368 (6,6)	122 (5,1)	1649 (7,0)	1597 (6,3)	
Privado	6952 (13,5)	319 (13,5)	4288 (18,0)	2345 (9,2)	
Admisión en UCI	3403 (6,6)	58 (2,5)	1748 (7,4)	1597 (6,3)	<0,001
Uso de ventilación mecánica	4073 (7,9)	83 (3,5)	2152 (9,1)	1838 (7,2)	<0,001
Periodo de infección					<0,001
Marzo/abril, 2020	5911 (11,5)	204 (8,6)	3072 (12,9)	2635 (10,4)	
Junio/julio, 2020	19 580 (38,0)	899 (38,0)	9033 (38,0)	9648 (38,0)	
Agosto/septiembre, 2020	19 027 (36,9)	816 (34,5)	8688 (36,5)	9523 (37,6)	
Octubre/noviembre, 2020	6985 (13,6)	447 (18,9)	2988 (12,6)	3550 (14,0)	
Fallecidos	22 787 (44,2)	200 (8,5)	6445 (27,1)	16 142 (63,7)	<0,001

a Valor de p calculado con la prueba de chi cuadrado. MINSA: Ministerio de Salud, EsSalud: Seguro Social de Salud del Perú, FFAA/PNP: Fuerzas Armadas y Policía Nacional, UCI: unidad de cuidados intensivos.

### Características según grupo de edad y mortalidad

Los varones fallecieron con más frecuencia a comparación de las mujeres (p<0,001). La mortalidad fue significativamente menor en los casos hospitalizados en un establecimiento privado en comparación con establecimientos vinculados a los servicios públicos (p<0,001). En los adultos, la mortalidad fue menor entre los casos que eran trabajadores de la salud (23,1%) en comparación de aquellos que no (27,6%, p<0,001). La mortalidad fue más frecuente entre los casos con comorbilidades, especialmente entre los jóvenes. Los jóvenes con alguna comorbilidad tuvieron tres veces (7,2% vs. 21,6%) la mortalidad de aquellos pacientes jóvenes sin comorbilidades (p<0,001). En los grupos de pacientes de adultos y adultos mayores, aquellos con comorbilidades también fallecieron con más frecuencia que aquellos sin comorbilidades, pero la diferencia fue menor que en los pacientes jóvenes. Tanto en el grupo de jóvenes, adultos y mayores, los casos con enfermedad cardiovascular, enfermedad neurológica crónica, enfermedad renal, enfermedad hepática o cáncer tuvieron mayor frecuencia de mortalidad en comparación con aquellos sin estas condiciones (p<0,05) (Tabla 2).

**Tabla 2:** Comparación de características entre fallecidos y supervivientes en pacientes con enfermedad por coronavirus 2019 confirmada que ingresaron a hospitalización en establecimientos de salud de Lima, Perú. Marzo a octubre del 2020.

Factores	Jóvenes (18-29)		Valor de p ^a^	Adultos (30-59)		Valor de p ^a^	Adultos mayores (≥60)		Valor de p ^a^
	Sobrevivientes n=2166	Fallecidos n=200		Sobrevivientes n=17336	Fallecidos n=6445		Sobrevivientes n=9214	Fallecidos n=16142	
	n (%)	n (%)		n (%)	n (%)		n (%)	n (%)	
Género			<0,001			<0,001			<0,001
Femenino	1347 (94,4)	80 (5,6)		6682 (77,0)	2000 (23,0)		4144 (40,3)	6146 (59,7)	
Masculino	819 (87,2)	120 (12,8)		10 654 (70,6)	4445 (29,4)		5070 (33,7)	9996 (66,3)	
Trabajador de salud			0,417			<0,001			0,756
No	1948 (91,4)	184 (8,6)		15 308 (72,4)	5836 (27,6)		8469 (36,3)	14 856 (63,7)	
Sí	218 (93,2)	16 (6,8)		2028 (76,9)	609 (23,1)		745 (36,7)	1286 (63,3)	
Comorbilidades			<0,001			<0,001			<0,001
No	2003 (92,8)	155 (7,2)		14 022 (75,5)	4540 (24,5)		5935 (37,7)	9822 (62,3)	
Sí	163 (78,4)	45 (21,6)		3314 (63,5)	1905 (36,5)		3279 (34,2)	6320 (65,8)	
Obesidad			0,192			<0,001			0,018
No	2083 (91,7)	188 (8,3)		16 387 (73,8)	5824 (26,2)		8892 (36,5)	15 480 (63,5)	
Sí	83 (87,4)	12 (12,6)		949 (60,4)	621 (39,6)		322 (32,7)	662 (67,3)	
Diabetes			0,036			<0,001			0,684
No	2142 (91,7)	194 (8,3)		16 072 (73,6)	5765 (26,4)		7835 (36,3)	13 758 (63,7)	
Sí	24 (80,0)	6 (20,0)		1264 (65,0)	680 (35,0)		1379 (36,6)	2384 (63,4)	
Enfermedad cardiovascular			<0,001			<0,001			<0,001
No	2150 (91,9)	190 (8,1)		16173 (73,6)	5794 (26,4)		7089 (37,0)	12 048 (63,0)	
Sí	16 (61,5)	10 (38,5)		1163 (64,1)	651 (35,9)		2125 (34,2)	4094 (65,8)	
Enfermedad neurológica crónica			<0,001			<0,001			<0,001
No	2160 (91,8)	194 (8,2)		17 255 (73,0)	6388 (27,0)		9120 (36,5)	15 850 (63,5)	
Sí	6 (50,0)	6 (50,0)		81 (58,7)	57 (41,3)		94 (24,4)	292 (75,6)	
Enfermedad renal			<0,001			<0,001			<0,001
No	2157 (91,9)	190 (8,1)		17 188 (73,3)	6269 (26,7)		9006 (36,7)	15549 (63,3)	
Sí	9 (47,4)	10 (52,6)		148 (45,7)	176 (54,3)		208 (26,0)	593 (74,0)	
Enfermedad pulmonar crónica			0,640			<0,001			<0,001
No	2152 (91,6)	198 (8,4)		17 188 (73,0)	6349 (27,0)		9066 (36,6)	15 703 (63,4)	
Sí	14 (87,5)	2 (12,5)		148 (60,7)	96 (39,3)		148 (25,2)	439 (74,8)	
Asma			0,742			0,199			0,305
No	2138 (91,6)	197 (8,4)		17069 (72,9)	6361 (27,1)		9113 (36,3)	15 988 (63,7)	
Sí	28 (90,3)	3 (9,7)		267 (76,1)	84 (23,9)		101 (39,6)	154 (60,4)	
Enfermedad hepática			0,085			<0,001			0,005
No	2162 (91,6)	198 (8,4)		17 287 (73,0)	6405 (27,0)		9166 (36,4)	16 006 (63,6)	
Sí	4 (66,7)	2 (33,3)		49 (55,1)	40 (44,9)		48 (26,1)	136 (73,9)	
Inmunodeficiencia			0,507			<0,001			0,554
No	2159 (91,6)	199 (8,4)		17 291 (73,0)	6392 (27,0)		9198 (36,3)	16 107 (63,7)	
Sí	7 (87,5)	1 (12,5)		45 (45,9)	53 (54,1)		16 (31,4)	35 (68,6)	
Cáncer			<0,001			<0,001			<0,001
No	2162 (92,0)	189 (8,0)		17 234 (73,2)	6304 (26,8)		9098 (36,6)	15 759 (63,4)	
Sí	4 (26,7)	11 (73,3)		102 (42,0)	141 (58,0)		116 (23,2)	383 (76,8)	
Tipo de establecimiento de salud			<0,001			<0,001			<0,001
MINSA	1353 (92,0)	118 (8,02)		6237 (65,1)	3347 (34,9)		3088 (33,2)	6210 (66,8)	
EsSalud	392 (86,3)	62 (13,7)		5891 (71,3)	2369 (28,7)		4113 (33,9)	8003 (66,1)	
FFAA/PNP	109 (89,3)	13 (10,7)		1259 (76,3)	390 (23,7)		533 (33,4)	1064 (66,6)	
Privado	312 (97,8)	7 (2,2)		3949 (92,1)	339 (7,9)		1480 (63,1)	865 (36,9)	
Admisión en UCI			<0,001			<0,001			<0,001
No	2134 (92,5)	174 (7,5)		16 679 (75,7)	5354 (24,3)		8930 (37,6)	14 829 (62,4)	
Sí	32 (55,2)	26 (44,8)		657 (37,6)	1091 (62,4)		284 (17,8)	1313 (82,2)	
Uso de ventilación mecánica			<0,001			0,000			<0,001
No	2122 (92,9)	161 (7,1)		16 563 (76,6)	5066 (23,4)		8900 (37,8)	14 618 (62,2)	
Sí	44 (53,0)	39 (47,0)		773 (35,9)	1379 (64,1)		314 (17,1)	1524 (82,9)	
Periodo de diagnóstico			0,182			<0,001			<0,001
Marzo/Abril, 2020	179 (87,7)	25 (12,3)		2242 (73,0)	830 (27,0)		990 (37,6)	1645 (62,4)	
Junio/Julio, 2020	821 (91,3)	78 (8,7)		6307 (69,8)	2726 (30,2)		3150 (32,6)	6498 (67,4)	
Agosto/Setiembre,2020	754 (92,4)	62 (7,6)		6361 (73,2)	2327 (26,8)		3558 (37,4)	5965 (62,6)	
Octubre/Noviembre, 2020	412 (92,2)	35 (7,8)		2426 (81,2)	562 (18,8)		1516 (42,7)	2034 (57,3)	

a Valor de p calculado con la prueba de chi cuadrado.

MINSA: Ministerio de Salud, EsSalud: Seguro Social de Salud del Perú, FFAA/PNP: Fuerzas Armadas y Policía Nacional, UCI: unidad de cuidados intensivos.

### Comorbilidades asociadas a la mortalidad

En el análisis de regresión ajustada la presencia de enfermedad neurológica, enfermedad renal, enfermedad hepática y cáncer se asoció a un mayor riesgo de mortalidad independientemente del grupo de edad. Adicionalmente, en casos jóvenes, la enfermedad cardiovascular (RR=2,16; IC 95%: 1,24-3,74) se asoció con mayor riesgo de mortalidad. En adultos, la obesidad (RR=1,30; IC 95%: 1,22-1,39), la diabetes (RR=1,12; IC 95%: 1,06-1,20), la enfermedad cardiovascular (RR=1,14; IC 95%: 1,07-1,21), la enfermedad pulmonar crónica (RR=1,30; IC 95%: 1,1-1,51) y la inmunodeficiencia (RR=2,18; IC 95%: 1,77-2,69) también fueron comorbilidades asociadas a la mortalidad. En los casos mayores, la obesidad (RR=1,12; IC 95%: 1,07-1,17) y la enfermedad pulmonar crónica (RR=1,13; IC 95%: 1,07-1,18) también fueron comorbilidades asociadas a la mortalidad. El asma no estuvo asociado a un mayor riesgo de mortalidad en el análisis bivariado o multivariado en ninguno de los grupos de edad (Tabla 3).

**Tabla 3:** Análisis de regresión de Poisson con varianza robusta para comorbilidades asociadas a mortalidad hospitalaria en pacientes con enfermedad por coronavirus 2019 admitidos en hospitalización en Lima, Perú. Marzo a octubre del 2020.

Comorbilidades	Modelo sin ajustar			Modelo ajustado ^a^		
	RR	IC 95%	Valor de p	RR	IC 95%	Valor de p
Edad 18-29 años (n=2366)						
Obesidad	1,53	0,88-2,63	0,130	0,75	0,45-1,26	0,282
Diabetes	2,41	1,16-4,99	0,018	1,46	0,72-2,96	0,296
Enfermedad cardiovascular	4,74	2,86-7,85	<0,001	2,16	1,24-3,74	0,006
Enfermedad neurológica crónica	6,07	3,39-10,85	<0,001	5,03	2,60-9,74	<0,001
Enfermedad renal	6,50	4,15-10,17	<0,001	5,01	2,88-8,71	<0,001
Enfermedad pulmonar crónica	1,48	0,40-5,46	0,553	0,86	0,31-2,40	0,777
Asma	1,15	0,39-3,39	0,804	0,51	0,24-1,10	0,087
Enfermedad hepática	3,97	1,27-12,42	0,018	3,82	1,16-12,55	0,027
Inmunodeficiencia	1,48	0,24-9,31	0,675	1,02	0,16-6,61	0,986
Cáncer	9,12	6,53-12,74	<0,001	7,29	4,56-11,64	<0,001
Edad 30-59 años (n=23781)						
Obesidad	1,51	1,41-1,61	<0,001	1,30	1,22-1,39	<0,001
Diabetes	1,32	1,24-1,41	<0,001	1,12	1,06-1,20	<0,001
Enfermedad cardiovascular	1,36	1,27-1,45	<0,001	1,14	1,07-1,21	<0,001
Enfermedad neurológica crónica	1,53	1,25-1,87	<0,001	1,36	1,12-1,65	0,002
Enfermedad renal	2,03	1,84-2,25	<0,001	1,87	1,68-2,09	<0,001
Enfermedad pulmonar crónica	1,46	1,25-1,71	<0,001	1,30	1,11-1,51	0,001
Asma	0,88	0,73-1,06	0,188	0,92	0,77-1,10	0,378
Enfermedad hepática	1,66	1,32-2,09	<0,001	1,55	1,21-1,99	0,001
Inmunodeficiencia	2,00	1,67-2,41	<0,001	2,18	1,77-2,69	<0,001
Cáncer	2,17	1,94-2,42	<0,001	2,38	2,10-2,70	<0,001
Edad >60 años (n=25356)						
Obesidad	1,06	1,01-1,11	0,011	1,12	1,07-1,17	<0,001
Diabetes	0,99	0,97-1,02	0,672	1,00	0,98-1,03	0,859
Enfermedad cardiovascular	1,05	1,02-1,07	<0,001	0,99	0,96-1,01	0,179
Enfermedad neurológica crónica	1,19	1,13-1,26	<0,001	1,10	1,03-1,16	0,002
Enfermedad renal	1,17	1,12-1,22	<0,001	1,10	1,06-1,15	<0,001
Enfermedad pulmonar crónica	1,18	1,12-1,24	<0,001	1,13	1,07-1,18	<0,001
Asma	0,95	0,86-1,05	0,296	0,98	0,89-1,07	0,613
Enfermedad hepática	1,16	1,07-1,27	0,001	1,20	1,10-1,31	<0,001
Inmunodeficiencia	1,08	0,90-1,30	0,427	1,14	0,96-1,37	0,144
Cáncer	1,21	1,15-1,27	<0,001	1,23	1,17-1,29	<0,001

a Ajustado por edad, sexo en años, ser personal de salud, ingreso a cuidado intensivos, necesidad de ventilación mecánica, tipo de establecimiento de salud y el periodo en el que se notificó el caso. RR: riesgo relativo, IC 95%: intervalo de confianza al 95%.

## DISCUSIÓN

En este estudio analizamos los datos de los casos adultos con COVID-19 hospitalizados durante la primera ola pandémica en Lima, Perú. Encontramos que la frecuencia de comorbilidades y mortalidad aumentó con la edad; las comorbilidades asociadas a la mortalidad, independientes del grupo de edad, fueron la enfermedad neurológica crónica, la enfermedad renal, la enfermedad hepática y el cáncer; y que el riesgo de mortalidad asociada con comorbilidades fue mayor en adultos jóvenes en comparación con los adultos y los adultos mayores. Hasta donde sabemos, este es el primer estudio que evalúa las comorbilidades asociadas con la mortalidad en diferentes grupos de edad. En Perú, estudios previos en escenarios locales, han reportado que la saturación de oxígeno al ingreso ^6^^,^^13^, el tener más de 65 años, un nivel de lactato deshidrogenasa mayor a 720 U/L ^13^ y el uso de la ventilación mecánica o el ingreso en la UCI ^14^, fueron factores asociados con la mortalidad.

La mortalidad en Lima y Callao fue mayor en comparación con el promedio mundial, estimado en 15% en una revisión sistemática ^15^, pero similar a otros países latinoamericanos como Colombia (40%) ^16^. Diferentes factores pueden llevar a que Perú tenga una de las tasas de mortalidad más altas del mundo. A ello contribuiría el colapso del sistema de salud por la rápida ocupación de camas de hospitalización y cuidados críticos, la falta de oxígeno y equipos de ventilación mecánica ^17^ y las altas tasas de automedicación con antibióticos o antiinflamatorios entre la población general que se estima que podría rondar el 39% entre los residentes de Lima ^18^.

Los pacientes mayores de 59 años tuvieron una mortalidad 20 veces mayor en comparación con los de 18 a 29 años. Los adultos jóvenes presentaron una mortalidad similar a la hallada en otro estudio en Estados Unidos ^19^. Los adultos mayores sufrieron una mayor mortalidad en comparación con otros países de ingresos altos (35,5%) ^20^, pero similar a estudios realizados en países de América Latina como Brasil (64%) ^21^ y México (69%) ^22^. La edad es uno de los factores de riesgo de mortalidad independiente, identificado desde el comienzo de la pandemia ^23^. Las personas mayores tienen menor reserva funcional y niveles más altos de la enzima convertidora de angiotensina 2 (ACE2), los que pueden tener un papel clave en la afectación multiorgánica provocada por el SARS-CoV-2 ^24^. De forma interesante, observamos que el riesgo de mortalidad asociado a comorbilidades fue mayor en pacientes jóvenes (18-29 años) en comparación con pacientes de mayor edad. Los pacientes más jóvenes con comorbilidades fallecieron tres veces más que los que pacientes jóvenes que no tenían comorbilidad, diferencia mucho mayor a la observada en los pacientes de mayor edad. También observamos en los modelos de regresión una asociación de mayor magnitud entre comorbilidades y mortalidad en pacientes más jóvenes que en pacientes mayores. Una potencial explicación es que los pacientes jóvenes podrían presentar comorbilidades de mayor severidad o complejidad que los pacientes de mayor edad llevando esto a un peor pronóstico al momento de tener la COVID-19 y sugiere que los jóvenes con comorbilidades podrían requerir un monitoreo particularmente cercano como grupo de alto riesgo.

La enfermedad neurológica crónica, la enfermedad renal, la enfermedad hepática y el cáncer se asociaron con la mortalidad, independientemente del grupo de edad. Este hallazgo concuerda con investigaciones previas. Por ejemplo, un estudio en España encontró que los pacientes previos con comorbilidades neurológicas preexistentes tienen 1,76 más probabilidades de morir, independientemente de la ocupación de camas o del tratamiento recibido ^25^. El estado de salud basal de estos pacientes podría ser una explicación, los pacientes con enfermedad neurológica crónica tienen mayor fragilidad y menor reserva que las personas sin enfermedad neurológica crónica. Las manifestaciones neurológicas durante la enfermedad aguda también se han asociado con un peor pronóstico ^26^. Una revisión sistemática mostró que los pacientes con enfermedad renal crónica tienen siete veces más probabilidades de morir que los pacientes sin enfermedad preexistente ^27^. Los pacientes con enfermedad renal tienen un estado proinflamatorio y defectos funcionales en las células inmunitarias innatas y adquiridas, lo que aumenta la vulnerabilidad a las infecciones y también empeora el pronóstico ^28^. Varios estudios además han demostrado que los pacientes con enfermedad renal crónica tienen un mayor riesgo de neumonía y neumonía grave ^29^. La enfermedad hepática también se ha asociado a una peor severidad y mortalidad ^30^. La obesidad también fue un factor de riesgo en el grupo de pacientes mayores de 30 años y esto concuerda con un metaanálisis que evidenció que los pacientes obesos tendrían hasta cuatro veces más mortalidad ^31^. No encontramos asociación entre los casos de 18 a 29 años. No observamos que el asma se asociara con un mayor riesgo de mortalidad. Este resultado también estuvo de acuerdo con la evidencia publicada. La OMS realizó una revisión sistemática que concluyó que el papel del asma como factor de riesgo de muerte por la COVID-19 sigue sin estar claro ^32^. Otra revisión encontró que el riesgo de infección era menor en las personas asmáticas a comparación de las no asmáticas, pero no observó diferencias en términos de hospitalización, admisión a cuidados intensivos, uso de ventilador o mortalidad ^33^.

Los resultados de este estudio deben considerar algunas limitaciones. En primer lugar, este es un análisis retrospectivo de fuentes datos secundarios (principalmente del sistema de vigilancia epidemiológica) que no fue diseñado para estimar factores de riesgo. Por esta razón, no incluye tantos detalles clínicos como lo haría la historia clínica electrónica. Encontramos una menor prevalencia de obesidad ^34^, enfermedad pulmonar obstructiva crónica ^35^ y enfermedad renal ^36^ que estudios con pruebas especializadas y lo asumimos a la falta de definiciones estandarizadas de comorbilidades en forma epidemiológica. Tampoco pudimos explorar la gravedad de las comorbilidades, que pueden modificar el efecto sobre la mortalidad. En segundo lugar, los hallazgos del estudio deben generalizarse con precaución a otras poblaciones debido a la situación caótica única que se vivió durante la primera ola pandémica en Perú, que puede no reflejar la realidad de todos los pacientes hospitalizados por la COVID-19. En tercer lugar, no disponemos de datos sobre variables como la saturación de oxígeno al ingreso o el tratamiento recibido durante la hospitalización, que podrían tener un papel importante en la mortalidad. En cuarto lugar, hubo pacientes diagnosticados únicamente con pruebas rápidas serológicas. Estas pruebas tienen un rendimiento diagnóstico menor que las pruebas moleculares, por lo que cabe la posibilidad de que algunos pacientes haya habido falsos positivos y falsos negativos. Por otro lado, este estudio tiene la fortaleza de analizar una muestra amplia y representativa de pacientes hospitalizados. Estudios previos en entornos locales no han explorado las comorbilidades asociadas con la mortalidad, por lo que nuestros resultados complementan el estudio de la COVID-19 en el Perú. Además, este es el primer estudio que evalúa las comorbilidades asociadas por grupos de edad. El análisis multivariado también consideró posibles factores de confusión, como la ocupación de camas en la UCI del hospital, el tipo de establecimiento de salud y el período desde el inicio de los síntomas hasta la hospitalización.

En conclusión, en esta cohorte retrospectiva de pacientes hospitalizados con la COVID-19, encontramos evidencia de que la enfermedad neurológica crónica, la enfermedad renal, la enfermedad hepática y el cáncer fueron las comorbilidades asociadas con la mortalidad independientemente del grupo de edad. Además, a pesar de que los pacientes mayores de 60 años tuvieron una mayor mortalidad en comparación con los pacientes más jóvenes, el riesgo de mortalidad asociada a las comorbilidades fue mayor en los adultos jóvenes.
